# The NF-κB1/p50 Subunit Influences the Notch/IL-6-Driven Expansion of Myeloid-Derived Suppressor Cells in Murine T-Cell Acute Lymphoblastic Leukemia

**DOI:** 10.3390/ijms25189882

**Published:** 2024-09-13

**Authors:** Behnaz Abdollahzadeh, Noemi Martina Cantale Aeo, Nike Giordano, Andrea Orlando, Maria Basciani, Giovanna Peruzzi, Paola Grazioli, Isabella Screpanti, Maria Pia Felli, Antonio Francesco Campese

**Affiliations:** 1 Department of Molecular Medicine, Sapienza University of Rome, 00161 Rome, Italy; behnaz.abdollahzadeh@uniroma1.it (B.A.); noemimartina.cantaleaeo@uniroma1.it (N.M.C.A.); nike.giordano@hotmail.com (N.G.); orlandoandrea88@gmail.com (A.O.); marbasciani97@gmail.com (M.B.); paolagrazioli122@gmail.com (P.G.); isabella.screpanti@uniroma1.it (I.S.); 2 Center for Life Nano- and Neuro-Science, Fondazione Istituto Italiano di Tecnologia (IIT), 00161 Rome, Italy; giovanna.peruzzi@iit.it; 3 Department of Experimental Medicine, Sapienza University of Rome, 00161 Rome, Italy; mariapia.felli@uniroma1.it

**Keywords:** Notch, T-ALL, MDSC, NF-κB1/p50, IL-6, tumor microenvironment

## Abstract

T-cell acute lymphoblastic leukemia is an aggressive neoplasia due to hyper-proliferation of lymphoid progenitors and lacking a definitive cure to date. Notch-activating mutations are the most common in driving disease onset and progression, often in combination with sustained activity of NF-κB. Myeloid-derived suppressor cells represent a mixed population of immature progenitors exerting suppression of anti-cancer immune responses in the tumor microenvironment of many malignancies. We recently reported that in a transgenic murine model of Notch3-dependent T-cell acute lymphoblastic leukemia there is an accumulation of myeloid-derived suppressor cells, dependent on both Notch signaling deregulation and IL-6 production inside tumor T-cells. However, possible interaction between NF-κB and Notch in this context remains unexplored. Interestingly, we also reported that Notch3 transgenic and NF-κB1/p50 deleted double mutant mice display massive myeloproliferation. Here, we demonstrated that the absence of the p50 subunit in these mice dramatically enhances the induction and suppressive function of myeloid-derived suppressor cells. This runs in parallel with an impressive increase in IL-6 concentration in the peripheral blood serum, depending on IL-6 hyper-production by tumor T-cells from double mutant mice. Mechanistically, IL-6 increase relies on loss of the negative control exerted by the p50 subunit on the IL-6 promoter. Our results reveal the Notch/NF-κB cross-talk in regulating myeloid-derived suppressor cell biology in T-cell leukemia, highlighting the need to consider carefully the pleiotropic effects of NF-κB-based therapy on the tumor microenvironment.

## 1. Introduction

T-cell acute lymphoblastic leukemia (T-ALL) represents an uncommon aggressive hematological malignancy, caused by oncogenic transformation of developing T-cell progenitors [1,2,3]. It includes about 10–15% of pediatric and about one-fourth of adult acute lymphoblastic leukemia patients, and, despite recent progress in the efficacy of conventional therapies, it remains an urgent clinical problem because of the poor survival rate in cases with relapsed disease (that represent the 40–75% of adult and the 15–20% of pediatric patients) [4,5].

The Notch pathway includes membrane receptors (Notch-1 to -4, in mammals) that interact with specific ligands (Jagged-1 and -2; Dll-1, -3, and -4, in mammals) to regulate gene expression via translocation of the intracellular domain (ICN) into the nucleus, where it acts inside a transcriptional complex in combination with the DNA-binding factor CSL/RBP-Jk and other co-factors [6,7]. Though essentially conserved in metazoans, the Notch pathway is also characterized by highly context-dependent outcomes. In cancer, Notch receptors could exert a role ranging from that of an oncogene to that of a tumor suppressor [8,9,10].

Notably, hyper-activating mutations of Notch-1 and/or Notch-3 are events occurring in the vast majority of T-ALL cases [11,12,13,14], reflecting the fact that the two receptors regulate many decision steps of T-cell development and differentiation [15,16,17,18]. Not surprisingly, Notch signaling has often represented a main target in the search of T-ALL therapies [19,20]. About 30 years of research, since the first association of Notch with ‘rare’ human T lymphoblastic neoplasms [21], have led to the description of a plethora of Notch signaling modulators inside tumor T-cells, mainly through the study of consolidated murine models of Notch-dependent T-ALL [22,23], but all of them remain necessary but not sufficient to the onset and/or progression of the disease [20]. Thus, it has acquired increasing importance to explore the role of the tumor microenvironment (TME) and of non-cell autonomous mechanisms possibly influencing behavior of T-ALL cells (T-ALLs), such as epigenetic regulation by miRNAs [24,25,26,27,28], chemokine-driven interactions with the stromal compartment [29,30,31,32], and immune-evasion strategies, including immunosuppression, through regulatory T-cells (Tregs) and myeloid-derived suppressor cells (MDSCs) [33,34,35].

MDSCs represent a heterogeneous group of immature/precursor cells induced by various pathways, including that of Notch, that are emerging as a new target of cancer immunotherapy [36,37,38,39]. Inside the TME of many solid and hematological tumors, MDSCs indeed suppress anti-tumor immune responses, especially those of T-cells and NK-cells, thus facilitating disease progression. In this context, we have recently demonstrated that the constitutive activation of Notch inside T-ALL cells, from both mice and humans, can induce the accumulation in trans of CD11b^+^GR-1^+^ MDSCs through a Notch/IL-6-dependent mechanism [40]. Further, induced MDSCs sustain, in turn, expansion and proliferation of tumor T-cells [40]. In our transgenic murine model of Notch-dependent T-ALL, the *N3tg* mice [23], harboring a lck proximal promoter-driven overexpression of the Notch3 intracellular domain (ICN), targeted to immature T-cells.

In the search of pathways regulating the Notch/IL-6-dependent induction of MDSCs in T-ALL, we start considering NF-κB, an inducible transcription factor that regulates crucial processes (such as differentiation, proliferation, and inflammation) in both physiological and pathological conditions [41]. In mammals, the NF-κB family is activated by several stimuli and delivers signals through two different pathways: the canonical one, which comprises the NF-κB1/p50, RelA (p65), and c-Rel subunits, and the non-canonical pathway, which relies on the NF-κB2/p52 and RelB proteins. In the classical scheme, NF-κB can regulate transcription at the level of many gene promoters through the binding of activating p50/p65 and p50/c-Rel heterodimers or inhibitory p50/p50 homodimers [41]. Moreover, NF-κB is one of the most important molecules linking inflammation to cancer [42]. Interestingly, it is activated not only inside cancer cells but also in different cell subsets of the TME. Thus, its final effect on the anti-tumor immune response could be ambivalent depending on the cellular context [43,44]. Finally, the intimate intersection between the Notch and NF-κB signaling pathways is well recognized in many contexts, including the immune system and pathogenesis of Notch-dependent T-ALL [23,45,46,47,48]. We previously demonstrated that the deregulated activation of Notch3 inside thymocytes and malignant T-cells of our *N3tg* transgenic murine model of T-ALL induces the constitutive activation of the NF-κB complex, mainly represented by the p50/p65 heterodimers, via IKKα-dependent degradation of IκBα. This activation promotes the enhancement of NF-κB-dependent anti-apoptotic and proliferative pathways, sustaining the survival of tumor T-cells [23].

In line with the issues highlighted above, we previously demonstrated that genetic deletion of the NF-κB1/p50 subunit in the Notch3 transgenic background deeply impacts the TME, inducing a dramatic expansion of the myeloid compartment in *N3tg*/*p50*^−/−^ double mutant mice, represented by CD11b^+^GR-1^+^ cells. This process induces the rise of a fatal myeloproliferative trait that is T-cell dependent [49]. Additionally, these mice display a significant delay of T-ALL progression with respect to the *N3tg* controls, as evidenced by the reduction in tumor CD4^+^CD8^+^ DP T-cell number, due to a cell autonomous enhancement of their apoptotic rate [49].

Based on the premises above, our aim was to characterize the function of CD11b^+^GR-1^+^ cells from the *N3tg*/*p50*^−/−^ model in order to suggest the role of NF-κB as a potential interactor of Notch signaling in regulating MDSC induction in T-ALL. Now, we report here that expanded myeloid cells from *N3tg*/*p50*^−/−^ mice are indeed functional MDSCs. We also suggest that the uncontrolled expansion of this subset in double mutant mice derives from an exacerbation in the production of IL-6 cytokines by immature tumor T-cells. At a molecular level, we observe an enhanced transcription of the IL-6 promoter in the absence of inhibitory p50/p50 homodimers. Collectively, our results suggest NF-κB as a modulator of Notch in influencing MDSC biology in T-ALL.

## 2. Results

### 2.1. CD11b^+^GR-1^+^ Myeloid Cells Expand Significantly in N3tg/p50^−/−^ Mice at an Initial Stage of the Disease

Previously, we observed that myeloid cells with a CD11b^+^GR-1^+^ phenotype are significantly increased in *N3tg* mice with respect to *wt* controls [40] and even more expanded in *N3tg*/*p50*^−/−^ double mutant mice [49]. These observations were made in mice at an advanced age (i.e., at an advanced stage of the disease). Further, we demonstrated in vitro and in vivo that this process depends on non-cell autonomous mechanism/s driven by the T-cell compartment [49].

**Figure 1:**
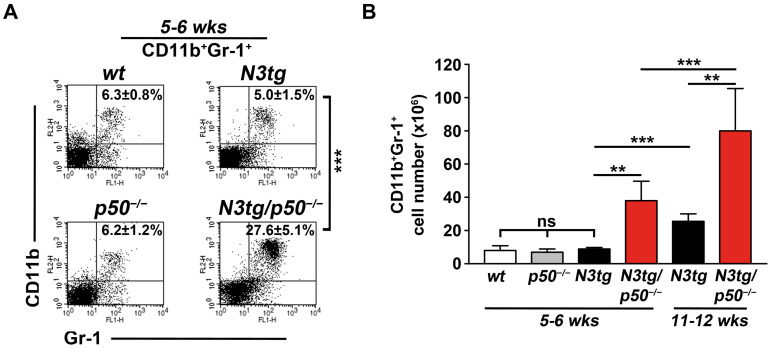
CD11b^+^GR-1^+^ cells accumulate in the spleen of *N3tg*/*p50*^−/−^ young mice. (**A**) Representative dot plots showing CD11b versus Gr-1 distributions in the spleen of *wt*, *p50*^−/−^, *N3tg*, and *N3tg*/*p50*^−/−^ mice at 5–6 weeks of age, as measured by FACS analysis. The numbers inside each cytogram represent percentages of CD11b^+^Gr-1^+^ cells. (**B**) CD11b^+^Gr-1^+^ numbers in the spleen from *wt*, *p50*^−/−^, *N3tg*, and *N3tg*/*p50*^−/−^ mice at 5–6 weeks of age (left part), as well as from *N3tg* and *N3tg*/*p50*^−/−^ mice at 11–12 weeks of age (right part), as assessed by FACS analysis, as in (**A**). Data represent mean ± SD of four independent experiments (*n* = 4 mice for each genotype and for each age). ** *p* ≤ 0.01 and *** *p* ≤ 0.001 represent significant differences between the indicated groups. ns = not significant, *p* > 0.05.

Here, we want to check for myeloproliferation in *N3tg*/*p50*^−/−^ and *N3tg* young mice, starting from an initial stage of the disease, when the secondary effects of the disease are likely reduced. FACS analysis in the spleen (Figure 1) evidenced that CD11b^+^GR-1^+^ cells are significantly increased in double-mutant mice with respect to *N3tg* littermates in percentages (Figure 1A; 27.6 ± 5.1% versus 5.0 ± 1.5%, respectively), as well as in absolute numbers (Figure 1B; 38.0 ± 11.8 × 10^6^ versus 8.9 ± 0.9 × 10^6^, respectively), at 5–6 weeks of age, and they increase progressively with age (Figure 1B, compare red bars at 11–12 versus 5–6 weeks of age: 80.1 ± 25.5 × 10^6^ and 38.0 ± 11.8 × 10^6^, respectively). No significant differences in absolute numbers of CD11b^+^GR-1^+^ cells were observed for both *wt* and *p50*^−/−^ controls at 11–12 weeks of age, with respect to 5–6 weeks of age [49]. More importantly, in the bar graph of Figure 1B, we also reported that no relevant differences were present between *N3tg* mice and *wt* or *p50*^−/−^ controls in young animals at 5–6 weeks of age and that the CD11b^+^GR-1^+^ subset starts expanding significantly in *N3tg* mice at later time points (Figure 1B, compare black bars at 11–12 versus 5–6 weeks of age: 25.5 ± 4.5 × 10^6^ and 8.9 ± 0.9 × 10^6^, respectively).

In summary, we confirm that myeloproliferation of CD11b^+^GR-1^+^ cells is dependent on Notch3 deregulation, but it appears in *N3tg* mice only at later time points, whereas it improves more intensively in the absence of the NF-κB1/p50 subunit in *N3tg*/*p50*^−/−^ double mutant mice, starting from an initial phase of the disease.

### 2.2. The Genetic Deletion of the NF-κB1/p50 Subunit in N3tg Mice Enhances the Suppressive Function of T-ALL-Induced CD11b^+^GR-1^+^ MDSCs

Recently, we published that in our *N3tg* murine model of Notch-dependent T-ALL, the expanded CD11b^+^GR-1^+^ cells are functional MDSCs, induced by the deregulation of Notch signaling inside T-ALL tumor T-cells through non-cell autonomous mechanisms [40]. These results prompted us to analyze in more detail features of CD11b^+^GR-1^+^ cells in *N3tg*/*p50*^−/−^ double mutant mice to assess if they could represent functional MDSCs.

**Figure 2:**
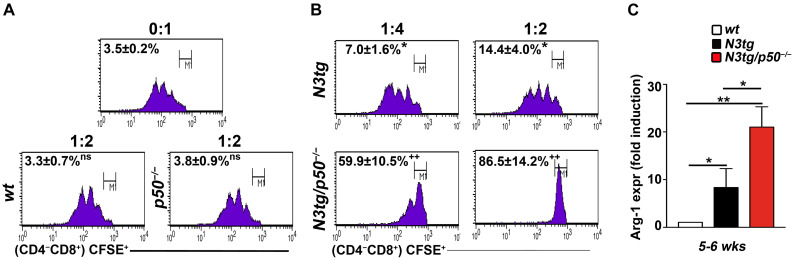
CD11b^+^GR-1^+^ cells from *N3tg*/*p50*^−/−^ mice are functional MDSCs. Representative FACS analysis at 72 h of an in vitro suppression assay with activated, CFSE-labeled *wt* T splenocytes, CD4^−^CD8^+^ gated, used as ‘responders’. In (**A**), *wt* T ‘responder’ cells were cultured alone, as a negative control (((**upper panel**), at a 0:1 suppressor/responder ratio), or in combination, with CD11b^+^GR-1^+^ cells from the spleen of *wt* or *p50*^−/−^ mice, at 5–6 weeks of age ((**lower panels**), at a 1:2 suppressor/responder ratio), as a control. In (**B**), ‘responders’ were co-cultured with CD11b^+^GR-1^+^ cells from the spleens of *N3tg* or *N3tg*/*p50*^−/−^ mice at 5–6 weeks of age ((**left panels**), at a 1:4 suppressor/responder ratio; (**right panels**), at a 1:2 suppressor/responder ratio). The numbers inside the cytograms represent the percentages of M1 non-proliferating, suppressed *wt* CD4^−^CD8^+^ T ‘responders’. The ratios of CD11b^+^GR-1^+^ ‘suppressors’: *wt* CD4^−^CD8^+^ T ‘target’ cells are also indicated above the panels (0:1, 1:4, or 1:2). (**C**) RT-qPCR assay of relative arginase-1 (Arg-1) mRNA expression in CD11b^+^GR-1^+^ cells purified from the spleens of *N3tg*, *N3tg*/*p50*^−/−^, and *wt* mice at 5–6 weeks of age. The expression level of Arg-1 mRNA in *wt* CD11b^+^GR-1^+^ controls was set to 1. Data represent the mean ± SD of three independent experiments (*n* = 3 mice for each genotype), each in triplicate. In (**A**,**B**), ns = not significant, *p* > 0.05, and * *p* ≤ 0.05 represents significant differences with respect to the negative control with *wt* T-cells cultured alone (the upper panel in (**A**)). ^++^ *p* ≤ 0.01 represents significant differences with respect to the *N3tg* counterparts (comparing the lower versus upper panels in (**B**)). In (**C**), * *p* ≤ 0.05 and ** *p* ≤ 0.01 represent significant differences between the indicated groups.

It is well established that the characteristic that serves to classify unequivocally myeloid cells as MDSCs is their suppressive function [36]. Thus, by an in vitro suppression assay, we tested the ability of CD11b^+^GR-1^+^ cells sorted from the spleen of *N3tg*/*p50*^−/−^ and *N3tg* young mice, as well as *wt* or *p50*^−/−^ littermates, at 5–6 weeks of age, to be ‘suppressors’ of the proliferation of *wt* spleen T-cells, previously marked with the ‘Carboxyfluorescein succinimidyl ester’ (CFSE) fluorescent dye (to measure their proliferation) and activated with anti-CD3/CD28 antibodies, and then used as ‘responders’ in co-cultures at the 1:4 or 1:2 (suppressor/responder) ratio (Figure 2).

FACS analysis at 72 h reveals that co-cultures with CD11b^+^GR-1^+^ splenocytes from *wt* controls or *p50*^−/−^ mice are both characterized by an unchanged rate of non-proliferating *wt* CD4^−^CD8^+^ T ‘responders’ (Figure 2A, lower panels: 3.3 ± 0.7% and 3.8 ± 0.9%, respectively, for the 1:2 ratio), compared to that observed in the negative controls, represented by activated *wt* CD4^−^CD8^+^ T-cells cultured alone (Figure 2A, upper panel: 3.5 ± 0.2%, for the 0:1 ratio). These results show that CD11b^+^GR-1^+^ cells are not functional MDSCs in *wt* mice (as expected) and, more importantly, in *p50*^−/−^ mice, where the NF-κB1/p50 subunit deletion does not confer any suppressive capacity to double mutant CD11b^+^GR-1^+^ cells at all. Instead, CD11b^+^GR-1^+^ cells from *N3tg*/*p50*^−/−^ mice exert a dose-dependent immunosuppressive function, even more potent than that of their *N3tg* counterparts (Figure 2B: 59.9 ± 10.5% versus 7.0 ± 1.6, respectively, for the 1:4 ratio in the left panels; 86.5 ± 14.2% versus 14.4 ± 4.0, respectively, for the 1:2 ratio in the right panels).

To corroborate the results above, we also considered levels of mRNA expression of the arginase-1 enzyme, which is highly expressed in MDSCs and linked to their suppressive function in cancer [50]. In purified CD11b^+^GR-1^+^ spleen cells, we reported a significant fold induction of Arg-1 mRNA expression in *N3tg* mice, that further increase in *N3tg*/*p50*^−/−^ animals, with respect to *wt* controls (Figure 2C), at 5–6 weeks of age.

Collectively, our observations demonstrate that the NF-κB1/p50 subunit deletion in *N3tg* young mice significantly increases suppressive function of Notch-induced CD11b^+^GR-1^+^ MDSCs.

### 2.3. The Enhanced Expansion of CD11b^+^GR-1^+^ MDSCs in N3tg/p50^−/−^ Double Mutant Mice Correlates with the Significant Increase in the IL-6 Cytokine

In our recent publication, we demonstrated that the accumulation of MDSCs driven by Notch signaling deregulation in our *N3tg* model depends on the IL-6 cytokine, which significantly increases in the peripheral blood serum of these mice at an advanced age and is primarily produced by CD4^+^CD8^+^ DP T-ALL cells [40].

**Figure 3:**
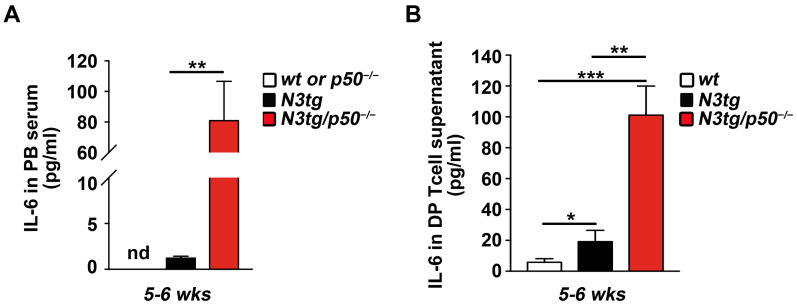
*N3tg*/*p50*^−/−^ mice display enhanced production of the IL-6 cytokine. IL-6 protein concentrations (pg/mL) assessed by ELISA (**A**) in peripheral blood *serum* from *N3tg*/*p50*^−/−^ and *N3tg* mice, at 5–6 weeks of age, compared to those of their *wt* or *p50*^−/−^ controls and (**B**) in the supernatant medium of CD4^+^CD8^+^ DP T splenocytes derived from *N3tg*/*p50*^−/−^ and *N3tg* mice, at 5–6 weeks of age, cultured alone for 48 h, compared to DP T thymocytes from their *wt* littermates. Data represent the mean values ± SDs of three independent experiments (*n* = 3 mice for each genotype), each in triplicate. nd, not determined; * *p* ≤ 0.05, ** *p* ≤ 0.01, and *** *p* ≤ 0.001 represent significant differences between the indicated samples.

Thus, we checked IL-6 protein levels in *N3tg*/*p50*^−/−^ double mutant mice (Figure 3), in search of its possible involvement in the dramatic expansion of this suppressor cell subset reported above for double mutant mice.

We noted that the IL-6 protein is virtually absent in the peripheral blood *serum* of both *wt* and *p50*^−/−^ controls at 5–6 weeks of age, whereas it reaches appreciable but low levels in *N3tg* mice and, notably, a considerable increase in *N3tg*/*p50*^−/−^ double mutant mice (Figure 3A).

Then, we cultured in vitro CD4^+^CD8^+^ DP T-cells, purified from the spleens of *N3tg* and *N3tg*/*p50*^−/−^ young mice, at 5–6 weeks of age, as well as from the thymuses of *wt* mice, as a control, alone and without any external stimuli, in order to check for the concentration of IL-6 released in the supernatant at 48 h (Figure 3B). We reported that *wt* DP T thymocytes produce a very low level of IL-6 (<6.0 pg/mL). Instead, the enhancement of the IL-6 protein released into the medium was significant in samples from *N3tg* mice and very impressive for cultures of DP T splenocytes from *N3tg*/*p50*^−/−^ double mutant mice, with respect to their relative controls.

In sum, we can conclude that an enhanced release of the IL-6 protein, as specifically documented for CD4^+^CD8^+^ DP T-cells, correlates with the early expansion of functional MDSCs in *N3tg*/*p50*^−/−^ young mice when compared to the *N3tg* counterpart.

### 2.4. The Genetic Ablation of the NF-κB1/p50 Subunit Enhances the Transcription of the IL-6 Promoter in CD4^+^CD8^+^ DP T-Cells from N3tg/p50^−/−^ Mice

In the attempt to elucidate at molecular level a mechanism that could explain the enhanced expression of the IL-6 cytokine and the increased accumulation of MDSCs in *N3tg*/*p50*^−/−^ double mutant mice, we start considering possible Notch-mediated alteration in transcriptional control of the IL-6 promoter. During the T-cell activation process, Notch is able to interact directly with NF-κB, facilitating its retention inside the nucleus and activity on the IFN-γ promoter [51]. In another context, it was reported that the NF-κB1/p50 subunit is a direct negative regulator of IL-6 promoter transcription in follicular B cells from *wt* mice, and its deletion could release this block, thus favoring an enhanced release of IL-6 in the microenvironment of *p50*^−/−^ aged mice [52].

**Figure 4:**
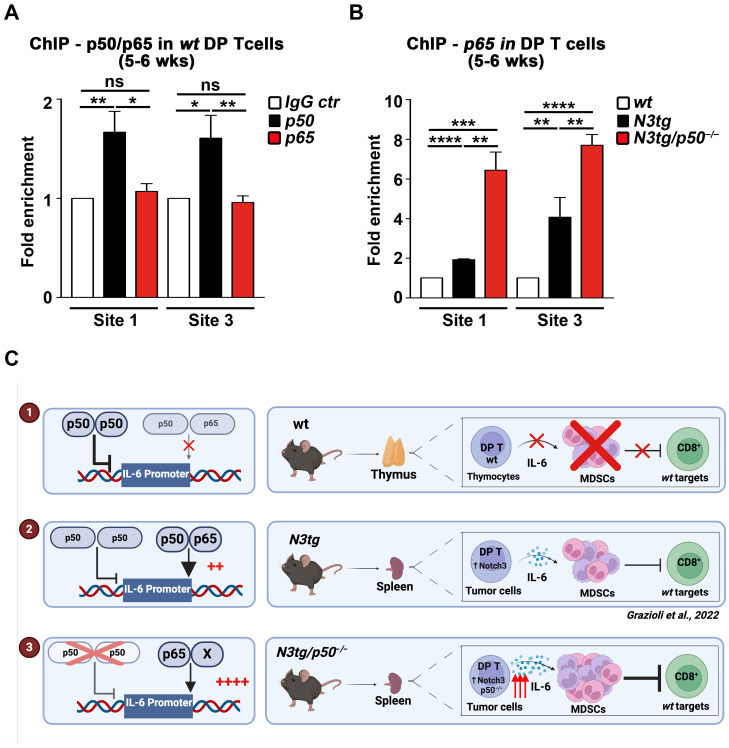
The NF-κB1/p50 subunit represses the transcription of the IL-6 promoter in CD4^+^CD8^+^ DP T-cells. (**A**) The binding of the NF-κB1/p50 or RELA/p65 subunit to the κB-consensus Site1 and Site3 of the murine IL-6 promoter [44], analyzed by ChIP-qPCR in cross-linked protein–DNA complexes of CD4^+^CD8^+^ DP T thymocytes from *wt* mice, at 5–6 weeks of age. Fold enrichment of target region in p50-IP, p65-IP, or control IgG-IP is shown, and data are normalized to binding at the β-actin promoter (negative control). The values of IgG-IP controls were set to 1. (**B**) The binding of the RELA/p65 subunit to Site1 and Site3 of the murine IL-6 promoter, as in (**A**), analyzed by ChIP-qPCR in cross-linked protein–DNA complexes of CD4^+^CD8^+^ DP T splenocytes from *N3tg* or *N3tg*/*p50*^−/−^ mice, compared to *wt* DP T thymocyte controls, at 5–6 weeks of age. Fold enrichment of target region in p65-IP versus control IgG-IP is shown, and data are normalized to binding at the β-actin promoter (negative control). The values in *wt* DP T thymocyte controls were set to 1. In (**A**,**B**), data represent the mean values ± SDs of three independent experiments (*n* = 3 mice for each genotype), each in triplicate. ns = not significant, *p* > 0.05. * *p* ≤ 0.05, ** *p* ≤ 0.01, *** *p* ≤ 0.001, and **** *p* ≤ 0.0001 represent significant differences between the indicated samples. (**C**) Working hypothesis on how the NF-κB1/p50 subunit can regulate IL-6 transcription and then MDSC differentiation in mice of different genotypes (see the text for an exhaustive explanation). In the left parts, x = no transcription, ++ = enhanced transcription and ++++ = very high transcription of the IL-6 promoter; vertical black arrows of different thickness indicate differences in the strength of p65 binding to the IL-6 promoter; blunt arrows of different thickness indicate differences in the inhibition of IL-6 transcription, that lacks in the panel 3 due to the absence of p50/p50 homodimers, as indicated by the red X over them. In the right parts, the triple red arrow indicates very high levels of IL-6 production by DP T cells; blunt arrows of different thickness indicate differences in the inhibition of CD8^+^ cells exerted by MDSCs, that lacks in the panel 1 due to the absence of IL-6 and MDSCs, as indicated by the red X over them. Created with BioRender.com.

On these premises, we tested the possible binding of the NF-κB1/p50 subunit at each of the four κB-consensus sites, previously described inside the murine IL-6 promoter [44], by a ChIP assay on samples of CD4^+^CD8^+^ DP T thymocytes from *wt* young mice at 5–6 weeks of age. As depicted in Figure 4A, the p50 subunit binds to Site 1 and Site 3 of the murine IL-6 promoter, whereas the binding of the RELA/p65 subunit is virtually absent. Thus, the prevalence of an inhibitory effect of the p50 subunit on IL-6 promoter transcription could account for the production of the IL-6 protein at a very low level, if any, as we reported above for the culture medium of *wt* DP thymocytes and blood *serum* from *wt* young mice (see Figure 3, above).

In Figure 4B, we explored in more detail the binding of the activatory p65 subunit to those sites of the IL-6 promoter in DP T-cells from the spleens of different genotypes at 5–6 weeks of age (i.e., at an early phase of T-ALL development). We observed an increase in p65 binding to both Site 1 and Site 3 in tumor DP T-cells from *N3tg* mice, that is in line with the constitutive activation of the NF-κB canonical pathway, originally described in tumor T-cells from the *N3tg* transgenic model [23], as well as with the significant increase in IL-6 protein levels described before with respect to their *wt* counterparts (see Figure 3B, above). Surprisingly, the genetic ablation of the NF-κB1/p50 subunit in the *N3tg* genetic background causes an even more sustained increase in the binding of the p65 subunit to those sites, which, again, corresponds to the dramatic enhancement of the IL-6 protein in DP T splenocytes and blood serum from *N3tg*/*p50*^−/−^ double mutant mice (see Figure 3, above).

In conclusion, as shown in our working model in Figure 4C, we suggest that under normal conditions, as for *wt* DP T-cells that are confined into the thymus (Figure 4C, upper panel, #1), the NF-κB1/p50 subunit represses transcription from the IL-6 promoter, and consequently, we do not observe any release of the IL-6 protein in the microenvironment and myeloid precursors do not differentiate into MDSCs. Instead, the increase in the NF-κB/p65 binding to the IL-6 promoter observed in DP T-ALL cells from *N3tg* mice (Figure 4C, middle panel, #2) stimulates the transcription of IL-6 and its release in the TME, sustaining the differentiation of functional MDSCs. Finally, the absence of negative regulation exerted by the NF-κB1/p50 subunit on the IL-6 promoter, as observed in DP T-ALL cells from *N3tg*/*p50*^−/−^ double mutant mice (Figure 4C, lower panel, #3), induces a further increase in the p65 binding to the IL-6 promoter. Eventually, this results in the enhancement of IL-6 transcription and release in the TME, thus improving the accumulation and function of MDSCs.

## 3. Discussion

The poor clinical outcomes of relapsing T-ALL patients have led us to consider the emerging role of Notch in the TME and cancer immunotherapy [53] as a new underexplored target of innovative approaches for the definitive cure of this aggressive T-cell leukemia. Focusing our attention on MDSCs for their crucial ability to impair anti-tumor immune responses, we have reported that the Notch/IL-6 axis inside tumor T-cells is necessary but not sufficient for the in trans induction of functional MDSCs in the TME of our murine model of Notch-dependent T-ALL, as well as in human PBMCs [40].

Now, in the attempt to individuate partners of Notch signaling in this context, we highlight in this paper the important role of its interaction with the NF-κB pathway. The genetic ablation of the NF-κB1/p50 subunit in T-cells of the *N3tg* background (i.e., the coexistence of the two mutations) significantly anticipates the accumulation and strongly improves the suppressive function of induced MDSCs in the TME of *N3tg*/*p50*^−/−^ double mutant mice with respect to the *N3tg* controls. Importantly, no ectopic expression of the Notch3 transgene was reported in the myeloid compartments of these mice [49], and any MDSC expansion or suppressive function was observed in *p50*^−/−^ single knock-out controls at all (see Figure 1 and Figure 2, above). The other group suggested, rather, that accumulation of the p50 subunit into the nucleus of myeloid cells positively controls MDSC differentiation and function, and its deficiency (as in the *p50*^−/−^ model) or exclusion from the nucleus impaired MDSC action in a cancer setting [54,55,56].

Interestingly, a role of the Notch or NF-κB signaling pathway, either positive or negative, in the differentiation and function of cancer-associated MDSCs was reported many times, as reviewed in [34,53,57]. However, very often, the suggested roles were intrinsic to MDSCs or their myeloid precursor, and they implicated cell autonomous mechanisms. Here, for the first time to our knowledge, we emphasize a cross-talk between Notch and the NF-κB1/p50 subunit inside tumor T-cells that improves MDSC induction and activity inside the TME of T-ALL in trans through a non-cell autonomous mechanism.

In the attempt to shed light on the process involved in Notch/NF-κB combined enhancement of MDSCs, we observed that it coincided with the considerable increase in the level of IL-6, which represents a major regulator of MDSC activity in cancer [58]. Indeed, we evidenced higher concentrations of IL-6 in both blood *serum* and supernatant of CD4^+^CD8^+^ DP T-cells cultured alone from *N3tg*/*p50*^−/−^ double mutant *versus N3tg* mice, whereas very low levels, if any, were observed in samples from *wt* and *p50*^−/−^ controls (see Figure 3). Of note, it is known that progressive and uncontrolled levels of IL-6 production by follicular B-cells led to the development of chronic inflammation and multiorgan autoimmunity in aging *p50*^−/−^ mice [52]. However, significant increase in systemic IL-6 and signs of inflammatory pathology became evident in *p50*^−/−^ mice only at 36 weeks of age, compared to *wt* controls [59]. On the contrary, in *N3tg*/*p50*^−/−^ mice, the improvement of MDSC accumulation and function, as well as high IL-6 concentration in the *serum*, were already present at a very early stage, at 5–6 weeks of age (see Figure 1, Figure 2 and Figure 3).

In further detail, we unveiled that the huge amount of IL-6 produced by DP T-cells from *N3tg*/*p50*^−/−^ mice depends at molecular level by the negative role exerted by the NF-κB1/p50 subunit on IL-6 promoter transcription (see Figure 4). This mechanism was firstly proposed in murine follicular B cells [52]. Of interest, following the original observation demonstrating that the NF-κB family of proteins is a key regulator of IL-6 transcription [60,61], a Notch/NF-κB cross-talk in this process was later described in murine macrophages [62]. In this manuscript, as depicted in our model (Figure 4C), we propose that in physiological conditions (as in *wt* DP T thymocytes), the p50 subunit binds two sites on the murine IL-6 promoter and inhibits the binding of the p65 subunit, which is normally included in transcriptional active heterodimers of NF-κB. In *N3tg* DP tumor T-cells, the constitutive activation of canonical NF-κB [23] allows a stronger binding of the p65 subunit to the IL-6 promoter, thus enhancing its transcription. At the opposite side, the absence of the NF-κB1/p50 subunit (as in *N3tg*/*p50*^−/−^ DP T-cells) does release the repression on the IL-6 promoter, further improving its transcription.

It is noteworthy that several polymorphisms have been defined that cause decreased expression of NF-κB1 in different human malignancies, such as epithelial tumors [63] and gastric cancer [64], as well as in autoimmune diseases [65]. Notably, a subset of diffuse large B-cell lymphomas (DLBCLs) can be characterized by a polymorphism in the NF-κB1 gene, with decreased expression of the p50 monomer, and constitutive activation of the NF-κB pathway, associated with higher levels of IL-6 in patients [66].

We are conscious that our study presents important limitations. First, the validation of our results through the use of NF-κB inhibitors in our model would represent a more definitive proof of the NF-κB role as modulator of MDSC functions. However, NF-κB signaling modulation, in addition to the impact in trans on MDSCs, as reported here, can have a high negative side effect *in cis* on the fitness of T-ALL tumoral cells [23,49], as well as on other TME cell subsets, including MDSCs (see [57] and references therein), with a high risk to obtain confounding results from this kind of experiment. Moreover, *N3tg*/*p50*^−/−^ mice develop a rapidly progressive myeloproliferative disease [49], which could limit in part the windows for the in vivo administration of NF-κB inhibitors. Second, though immature CD4^+^CD8^+^ DP T tumor cells are one of the most represented cell subsets in the spleen and bone marrow of *N3tg*/*p50*^−/−^ young mice [49], we cannot exclude that other cell types (including MDSCs themselves) contribute to IL-6 production and release in the TME, thus sustaining the establishment of an immunosuppressive environment.

Finally, to complete the picture of the regulation of IL-6 promoter transcription in T-ALL cells, it remains to specify the possible role of the alternative pathway of NF-κB, as well as of other subunits, such as c-Rel, in the absence of the NF-κB1/p50 subunit. Certainly, all these aspects need to be explored in future studies.

Overall, we believe that our data contribute to delineating NF-κB as an important partner of Notch in driving MDSC induction inside the TME of T-ALL. They may offer the rationale for testing in our pre-clinical setting a combined therapy aiming to hit proliferation and survival of T-ALL cells but also to inhibit the induction of immunosuppression by using highly selective NF-κB inhibitors (such as p65 subunit inhibitors). Additionally, our conclusions represent, on the one hand, a strong premise for including in future analysis of human T-ALL patients the potential influence on the immune environment exerted by the combined deregulation of the Notch and NF-κB signaling pathways inside tumor T-cells. The balance between activating p50/p65 heterodimers and inhibitory p50/p50 homodimers on the IL-6 promoter of T-ALL cells could be explored as a potential prognostic factor in patients. On the other hand, our observations reinforce once again the concern, already reported in other cancer settings [44,67], that the design and testing of a multitarget therapy for Notch-dependent T-ALL, focused on NF-κB, should carefully consider the pleiotropic effects of systemic and unspecific NF-κB inhibitors, such as the IKK inhibitors [68,69,70,71]. The therapeutic use of any NF-κB inhibitor should be evaluated not only for its effectiveness on tumor T-cells but also for its possible side effects on MDSCs.

In this context, we now cannot avoid taking seriously into account the consequences on MDSCs and other immune cell subsets of the T-ALL tumor microenvironment.

## 4. Materials and Methods

### 4.1. Mice

The generation and typing of *N3tg* mice have been described elsewhere [23]. The *N3tg*/*p50*^−/−^ mice have been generated by the intercross between *p50*^−/−^ [72] and *N3tg* mice, as previously described [49]. *N3-tg*, *N3tg*/*p50*^−/−^, *p50*^−/−^, and *wt* mice were all maintained on a C57BL/6 background. Mice were bred and housed in the institute’s animal care facilities. Mice were monitored daily and euthanized when they displayed excessive discomfort. All experiments involving animals described in this study were conducted in compliance with the animal welfare Italian National Laws (D.lgs. 116/1992 and 26/2014) and were approved by the local Animal Welfare Committee at Sapienza University and by the Italian Ministry of Health (authorization protocol #1/2012 and #C1386.17).

### 4.2. Cell Culture

CD4^+^CD8^+^ DP T-cells were sorted, as described from the spleens of *N3tg* and *N3tg*/*p50*^−/−^ mice, as well as from the thymuses of *wt* controls, and were cultured in 6-well plates (2 × 10^6^ cells/well). All the cell culture samples were cultured at 37 °C and 5% CO_2_ in complete medium, that is, RPMI-1640 medium (GIBCO, ThermoFisher, Waltham, MA, USA), supplemented with 10% FBS, 10 U/mL penicillin and streptomycin, and 2 mM glutamine. At 48 h, the supernatants from culture samples were collected by centrifugation at 1000× *g* for 15 min at +4 °C, and assayed for IL-6 concentration by ELISA.

### 4.3. Flow Cytometry and Cell Sorting

Freshly isolated cell samples from the spleen and/or thymus of *N3tg*, *N3tg*/*p50*^−/−^, *p50*^−/−^, and *wt* mice were resuspended in PBS 1×, 2% FBS, and after erythrocyte lysis with ammonium chloride–potassium buffer, they were stained with surface marker detection for 30 min on ice, using anti-CD4-PerCPCy5.5 (RM4-5), anti-CD8-APC (53-6.7), anti-CD11b-FITC (M1/70), and anti-Gr-1-PE (RB6-8C5) antibodies (all from BD Bioscience, La Jolla, CA, USA). Samples were run on a FacsCalibur (BD Bioscience, La Jolla, CA, USA) and analyzed with CellQuest Pro v6.0 software (BD Bioscience, La Jolla, CA, USA).

For FACS-assisted cell sorting experiments [49], cell suspensions from the spleen and/or thymus of *N3tg*, *N3tg*/*p50*^−/−^, *p50*^−/−^, and *wt* mice were stained with anti-CD11b, anti-Gr-1, anti-CD4, and anti-CD8 antibodies, as above, to isolate CD11b^+^Gr-1^+^ putative MDSCs and/or CD4^+^CD8^+^ double-positive (DP) T-cells with a purity ≥98%, using a FACSAriaIII sorter equipped with BD FACSDiva v6.1.3 software (both from BD Bioscience, La Jolla, CA, USA).

### 4.4. In Vitro Suppression Assay with Murine MDSCs

To assay the inhibitory function of putative MDSCs, the suppression assay was performed, as previously described [40]. Briefly, total *wt* T splenocytes were used as the target, after isolation by negative selection with the Pan T Cell Isolation Kit II (Miltenyi Biotec, Bergisch Gladbach, Germany) and staining with 2.5 μM CFSE (Sigma Aldrich, St. Louis, MO, USA), which allows measurement of the proliferation rate of the labeled cells. A total of 3.0 × 10^5^ of CFSE-labeled *wt* T-cells (‘responders’) were activated with coated 3 μg/mL anti-CD3 and with 2 μg/mL of soluble anti-CD28 (both from BD Bioscience, San Diego, CA, USA) and were co-cultured with graded numbers of CD11b^+^Gr-1^+^ cells (‘suppressors’), sorted from the spleens of *N3tg*, *N3tg*/*p50*^−/−^, *wt*, or *p50*^−/−^ mice, as described above. Co-cultures were performed for 72 h in 96-well plates; then, samples were collected and appropriately stained, and inhibition of proliferation of CFSE-labeled T-cells was assessed by FACS analysis on gated CD4^−^CD8^+^ T subsets.

### 4.5. ELISA

Samples were obtained from culture supernatants, as described above, or from the *serum* of mice with different genotypes. For *serum* isolation, whole blood was allowed to clot for 20 min at RT, and supernatant (*serum*) was collected after centrifugation at 1000× *g* for 15 min at +4 °C. All samples were stored at −80 °C. IL-6 concentrations were assayed, in triplicates, by using the mouse IL-6 Quantikine ELISA kit (R&D Systems, Minneapolis, MN, USA), following the manufacturer’s instructions.

### 4.6. Chromatin Immunoprecipitation Assay (ChIP-Assay)

The chromatin immunoprecipitation assay (ChIP-Assay) was carried out through the use of the “EZ Chip Chromatin Immunoprecipitation Kit” (17-371, Sigma-Aldrich, St. Louis, MO, USA), following the manufacturer’s instructions. Briefly, samples of CD4^+^CD8^+^ DP T-cells from the spleen of *N3tg* and *N3tg*/*p50*^−/−^ mice, as well as from the thymus of *wt* controls, at 5–6 weeks of age, sorted as above, were treated with formaldehyde at a final concentration of 1% in order to “cross-link” protein complexes to DNA within the living nuclei. Then, chromatin immunoprecipitation was carried out with 5 μgr of antibodies against NF-κB1 p50 (E-10, sc-8414x, Santa Cruz Biotechnology, Santa Cruz, CA, USA), ChIP-grade antibodies against NF-κB p65/RelA (17-10060, Millipore, Burlington, MA, USA), or an equivalent amount of normal-mouse IgG (12-371, Sigma-Aldrich) as a negative control. To measure the relative enrichment on the four κB-consensus regions of the murine IL-6 promoter, SYBR-greeen qPCR was performed with the specific primers listed below, as reported in [52]. Reactions were performed in triplicate. Data were normalized to binding at the β-actin promoter.

1. IL-6/NF-κB Site1: Fw 5′-TGGTAAATACAGAGCATTTGGGTG-3′;
2. Rv 5′-TTGGGATAAAGTTGAGACAGGCT-3′;
3. IL-6/NF-κB Site2: Fw 5′-AGCCATTGCCCCCAGGAT-3′;
4. Rv 5′-GCACATATGTAGCAGAGGACTGT-3′;
5. IL-6/NF-κB Site3: Fw 5′-CCTCTTCCCTGGGGTCTCA-3′;
6. Rv 5′-TCAGAAGTCTCAACTAACCTGGAC-3′;
7. IL-6/NF-κB Site4: Fw 5′-GGGGTTTCCAACTTCAGTCCA-3′;
8. Rv 5′-AGTTGGTCCAATGACTAGCCC-3′.

### 4.7. RNA Isolation and RT-qPCR of Arginase-1

CD11b^+^Gr-1^+^ cells from the spleen of *N3tg*, *N3tg*/*p50*^−/−^ and *wt* mice, at 5–6 weeks of age, were purified, as above. Total RNA samples were extracted with TRIzol reagent (Invitrogen, Waltham, MA, USA). Reverse transcription was performed with the High-Capacity cDNA Reverse-Transcription Kit, and the expression of murine Arginase-1 (Mm00475988_m1 assay) was determined by TaqMan quantitative real-time RT-PCR, using the StepOn ePlus™ Real-Time PCR System (all from ThermoFisher, Waltham, MA, USA), by following instructions from the manufacturer. Data were analyzed by the DDCt method; murine HPRT was used as a reference.

### 4.8. Statistical Analysis

Results are presented as mean values ± SDs (standard deviations). Statistical significance was assigned by a two-tailed Student’s *t*-test (performed with GraphPad Prism v.7.0a software, San Diego, CA, USA). A value of *p* ≤ 0.05 was assumed as indicative of a significant difference between groups. All data shown are representative of at least three independent experiments, meaning a total of *n* = 3 mice for each genotype and for each age where appropriate, unless otherwise specified. The number of used mice is also reported in each figure legend. Technical triplicates were performed where appropriate, as indicated in the relative figure legend.

## Data Availability

The raw data supporting the conclusions of this article will be made available by the authors on request.
